# Motor neurons integrate cholinergic inputs through spatial organization of diverse nicotinic receptors

**DOI:** 10.1093/pnasnexus/pgag173

**Published:** 2026-05-20

**Authors:** Ankura Sitaula, Komal Kaur, Arianna Mogharrabi, Lizzy Olsen, Aref Zarin

**Affiliations:** Biology Graduate Program, Texas A&M University, College Station, TX 77843-3258, USA; Biology Graduate Program, Texas A&M University, College Station, TX 77843-3258, USA; Department of Biology, Texas A&M University, College Station, TX 77843-3258, USA; Biology Undergraduate Program, Texas A&M University, College Station, TX 77843-3258, USA; Biology Graduate Program, Texas A&M University, College Station, TX 77843-3258, USA; Texas A&M Institute for Neuroscience, Texas A&M University, College Station, TX 77843-3474, USA; Department of Biology, Texas A&M University, College Station, TX 77843-3258, USA; Texas A&M Institute for Neuroscience, Texas A&M University, College Station, TX 77843-3474, USA

**Keywords:** nAChR subunits, motor neurons, cholinergic transmission, *Drosophila* locomotion, acetylcholine

## Abstract

Neural circuit function depends not only on synaptic connectivity but also on the molecular composition and subcellular organization of neurotransmitter receptors. Here, we examine the expression, localization, and functional relevance of nicotinic acetylcholine receptors (nAChRs), the primary mediators of fast excitatory transmission in the *Drosophila* central nervous system (CNS). Functional nAChRs are pentamers assembled from 10 subunits (α1–α7 and β1–β3), yet how this diversity is deployed within defined circuits remains poorly understood. Using T2A-Gal4 reporters and endogenous protein tagging, we identify eight nAChR subunits (α1–α3, α5–α7, β1, and β2) expressed in larval motor neurons (MNs). MN-specific knockdown of individual subunits produces impairments in crawling, peristaltic timing, and protopodium dynamics, demonstrating that multiple nAChR subtypes contribute to motor output. Colocalization analyses reveal a wide range of spatial relationships, identifying subunit pairs with high, intermediate, and low overlap within MN dendritic and postsynaptic domains. Across subunit combinations, spatial organization correlates with pair-specific functional interactions: spatially segregated pairs tend to produce stronger locomotor defects when knocked down together, suggesting largely nonredundant contributions to cholinergic excitation of MNs, whereas highly colocalized pairs often show limited additional impairment. Notably, some colocalized pairs also exhibit additive effects, indicating that spatial proximity alone does not fully predict functional interaction. Dual knockdown of selected subunit pairs also reduces muscle contraction amplitude, linking receptor organization to motor output at the effector level. Together, these results indicate that MNs deploy multiple nAChR populations whose spatial arrangement shapes how cholinergic inputs contribute to locomotor output.

Significance statement Movement depends on how nerve cells activate muscles but this process is shaped not only by which cells are connected but also by the types of receptors they use to receive signals. We show that motor neurons in fruit fly larvae use several different nicotinic acetylcholine receptor subunits that are organized into distinct receptor populations within individual neurons, even though their dendrites are densely intermingled. Disrupting specific receptor combinations changes how well the animals crawl and how strongly their muscles contract. These findings reveal that receptor organization inside single neurons plays an important role in how chemical signals are translated into movement, providing insight into how molecular diversity supports brain function and behavior.

## Introduction

With advances in electron microscopy (EM)-based connectomics, we now have detailed wiring diagrams of neural circuits across species (1–4). These circuits comprise neurons with diverse neurotransmitter identities, each potentially expressing a broad array of neurotransmitter receptors (NRs) with distinct properties and subcellular localizations. This receptor diversity enables circuits to generate varied responses to the same neurotransmitter. To fully interpret connectomes and understand circuit function, it is essential to map the distribution and roles of NRs within these networks. However, how receptor diversity is organized and utilized within identified circuits remains largely unknown. To address this gap, we leverage the powerful genetic tools and high-resolution EM connectome available for *Drosophila* motor circuits to investigate the diversity and function of nicotinic acetylcholine receptors (nAChRs) in larval MNs.

Acetylcholine (ACh) acts through both ionotropic and metabotropic receptors to mediate fast and slow signaling, respectively. nAChRs, the ionotropic ACh receptors, are members of the cys-loop superfamily of pentameric ligand-gated ion channels and serve as central components of the cholinergic system (5). A defining feature of nAChRs is their molecular complexity, which gives rise to extensive structural and functional diversity. In *Drosophila*, 10 nAChR subunits—seven *α* and three *β*—are encoded, compared with 10 *α* and four *β* subunits in vertebrates (6–12). These subunits assemble into homo- or heteropentameric cation channels that mediate fast excitatory transmission. Each mature pentamer likely exhibits distinct expression patterns, channel properties, and regulatory mechanisms. However, the functional logic of this subunit diversity—and how it contributes to receptor heterogeneity within neural circuits—remains poorly understood in vivo.

Disruptions in the cholinergic system have been implicated in a range of human neurological and psychiatric disorders, including Alzheimer's and Parkinson's diseases, depression, autism, attention-deficit/hyperactivity disorder (ADHD), epilepsy, and schizophrenia (13–15). As central components of this system, nAChRs are promising targets for drug development aimed at treating these conditions (15). In addition, insect nAChRs are the primary targets of widely used agricultural insecticides (8, 10, 12), though resistance to these compounds has emerged in many insect populations (16). A deeper understanding of nAChR expression and function is therefore critical not only for advancing therapeutic strategies for human diseases but also for designing more selective and sustainable insecticides with minimal off-target effects on beneficial species, including humans. While heterologous systems such as *Xenopus laevis* oocytes have been invaluable for studying nAChR structure and function, they do not fully capture receptor behavior in native neuronal environments (17–19). Receptors expressed in these systems may differ from endogenous receptors due to differences in lipid composition, pre- and posttranslational modifications, and codon usage, all of which can influence receptor localization, assembly, and function (17). As a result, direct circuit-level in vivo studies remain essential yet scarce, leaving key aspects of nAChR biology unresolved.

*Drosophila* offers several key advantages for studying the distribution and function of nAChRs in the nervous system. Powerful genetic tools now allow endogenous tagging of nAChR subunits within their native genomic context (10, 20–23), avoiding artifacts associated with overexpression systems, such as aberrant protein accumulation or mislocalization. In parallel, high-resolution EM connectomes for both larval and adult *Drosophila* provide an ideal framework for mapping endogenously tagged nAChR subunits onto known neural circuits (24–27). nAChRs mediate excitatory neurotransmission within the *Drosophila* brain and are essential for neural development, synaptic plasticity, and cognitive activities such as learning and memory (28). Like their mammalian counterparts, *Drosophila* nAChRs also regulate dopamine release, supporting a conserved role in dopaminergic signaling (29, 30). Importantly, individual subunits contribute to specific biological processes. Temporal regulation of α1, α5, α6, and α7 supports maturation of cholinergic synapses (23, 31, 32). Null mutants of several nAChR subunits display diverse behavioral and morphological phenotypes, indicating pleiotropic roles beyond neurotransmission. For example, α1, α2, α3, and α6 null mutants exhibit reduced climbing ability and decreased adult longevity, while α1, α2, α5, and β3 mutants show curled abdomens, and α1, β1, and β3 mutants exhibit defective wing inflation (10, 33–35). Additionally, α7 is essential for the escape reflex via the giant fiber system (36), and α1 and α3 are involved in complex behaviors such as courtship and sleep (37–39). As these null mutants lack receptor expression throughout the animal, tissue-specific manipulations are critical to uncover subunit functions within defined neural populations. Together, these findings highlight the diverse roles of nAChRs in behavior, yet their circuit-level functions—particularly within motor circuits driving locomotion—remain largely unexplored.

To address these questions, we examined the expression, localization, and functional relevance of nAChR subunits in *Drosophila* larval motor neurons (MNs), which receive extensive cholinergic input from premotor interneurons. Using T2A-Gal4 reporters and endogenous protein tagging, we identified multiple nAChR subunits expressed in individual MNs and mapped their distribution at synaptic resolution. MN-specific RNAi knockdown revealed that many subunits are required for normal locomotion. We further combined colocalization analysis of endogenously tagged subunits with single and double knockdown experiments to assess whether spatial organization of subunits within MN dendrites correlates with functional outcomes. These experiments do not resolve how individual receptor populations encode specific locomotor parameters, but instead test whether spatially organized receptor diversity within single neurons has measurable consequences for motor output.

## Results

### nAChR subunits are widely expressed in larval MNs

**Figure 1:**
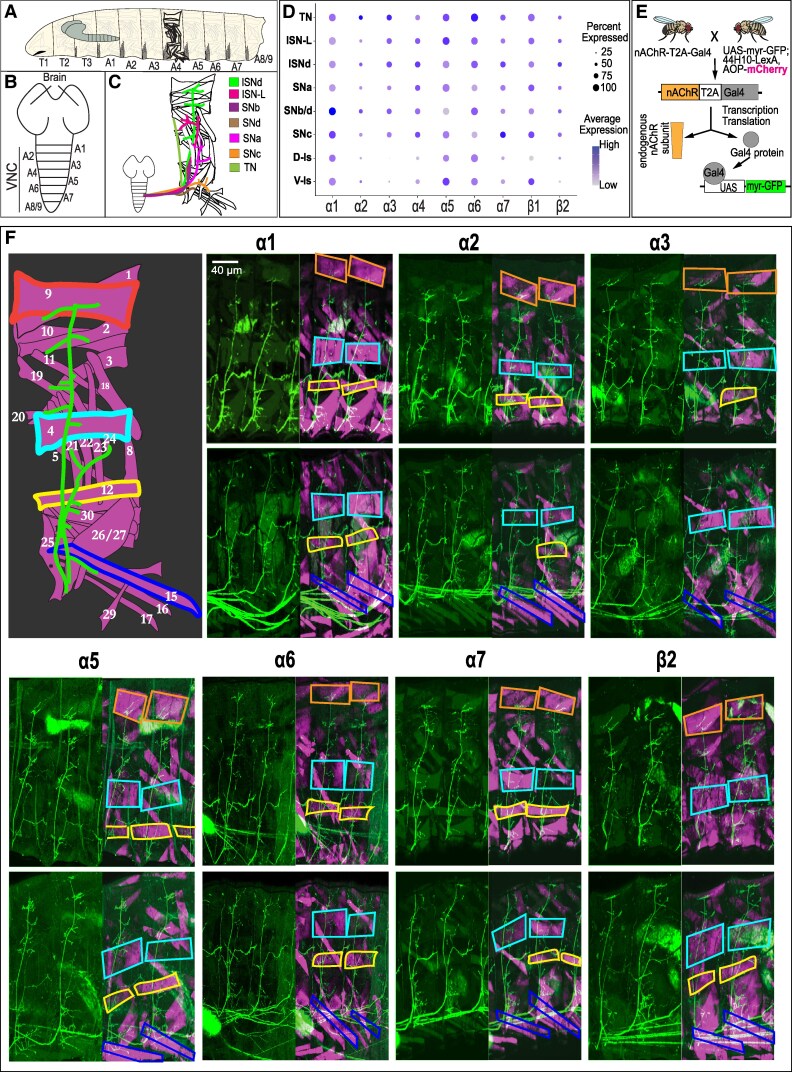
Larval MNs co-express a diverse repertoire of nAChR subunits. A) Schematic of the *Drosophila* larval body, composed of three thoracic and nine abdominal segments. B) Anatomy of the CNS, showing two brain lobes and the segmented VNC controlling body segments. C) Each abdominal (A1–A6) hemisegment contains ∼30 muscles, innervated by ∼30 excitatory glutamatergic MNs projecting via distinct nerve routes (color coded): ISNd, ISN-L, SNa, SNb, SNc, SNd, and TN. D) Dot plot showing nAChR subunit expression across MN clusters, based on scRNA-seq data from Nguyen et al. (40). Dot size reflects the percentage of expressing cells; color intensity reflects average expression level. E) nAChR-T2A-Gal4 reporter system schematic: T2A-mediated ribosomal skipping enables independent translation of Gal4 and the endogenous nAChR subunit. Gal4 then drives expression of UAS-myr-GFP to visualize nAChR-expressing MNs. F) Live imaging of expression patterns of seven nAChR subunits (α1–α3, α5–α7, and β2) in MNs, visualized using the strategy in (E). UAS-myr-GFP labels nAChR-expressing MNs, and muscles are labeled with GMR44H10-driven mCherry. The schematic indicates representative muscles are innervated by motor nerve bundles within each hemisegment. myr-GFP driven by different nAChR-T2A-Gal4 lines labels the motor axons corresponding to the nerve bundles shown in the schematic. Because the larval body wall is approximately cylindrical, two focal planes are shown for each subunit (dorsal-most to ventral and ventral-most to dorsal) to visualize motor axons throughout the imaging volume.

The *Drosophila* larval body is composed of three thoracic segments (T1–T3) and nine abdominal segments (A1–A9), with the corresponding ventral nerve cord (VNC) organized into 12 segmentally aligned neuromeres (Fig. 1A). The larval CNS consists of two brain lobes, a subesophageal zone, and a segmented VNC, which functions analogously to the vertebrate spinal cord. Each VNC segment controls the muscles of one body segment (Fig. 1B). Each abdominal segment contains ∼30 bilaterally symmetrical muscle pairs (Fig. 1C), innervated by a similar number of glutamatergic excitatory MNs, projecting from the corresponding VNC segment (41–43). These MNs exit the VNC through several nerve routes, including ISNd, ISN-L, SNb, SNa, SNc, and TN (Fig. 1C (44–47)). Based on firing properties and synaptic morphology, larval MNs are broadly categorized into two classes: phasic type Is MNs, which typically innervate multiple muscles and form small synaptic boutons, and tonic type Ib MNs, which innervate one to three muscles and form larger boutons at the neuromuscular junction (NMJ) (45–57).

All larval MNs receive excitatory cholinergic input from pre-MNs (pre-MNs), indicating that cholinergic receptors are essential components of the MN postsynaptic compartment (24, 58, 59). Recent single-cell RNA sequencing (scRNA-seq) of the larval VNC further supports this (40), revealing widespread expression of nAChR subunit transcripts across MNs belonging to the ISNd, ISN-L, SNb, SNa, SNc, and TN motor nerve bundles (Fig. 1D). However, the expression of individual subunits at the level of single, identified MNs has not been systematically characterized.

To address this gap, we used T2A-Gal4 lines for each nAChR subunit (60–62) in combination with upstream activation sequence (UAS)-membrane-targeted myristoylated green fluorescent protein (myr-GFP) to visualize subunit-expressing MNs. Postsynaptic muscles were simultaneously labeled using 44H10-LexA > LexAop-mCherry, enabling identification of individual MNs based on their target muscle(s) (Fig. 1E). This strategy allowed tracing of motor axon terminals and NMJs in intact larvae, permitting assessment of subunit expression at single-MN resolution. Using this approach, myr-GFP driven by different nAChR-T2A-Gal4 lines labeled axon bundles corresponding to ISNd, ISN-L, SNb, SNa, SNc, and TN. These data indicate that at least seven nAChR subunits (α1–α3, α5–α7, and β2) are expressed in the majority of larval MNs (Fig. 1F). For example, MN1, which innervates the most dorsal body wall muscle (muscle 1), expressed all seven of these subunits based on nAChR-T2A-Gal4 > myr-GFP labeling. These findings demonstrate broad and combinatorial expression of nAChR subunits across identified MNs, suggesting that multiple receptor subtypes contribute to cholinergic signaling within the motor system.

**Figure 2:**
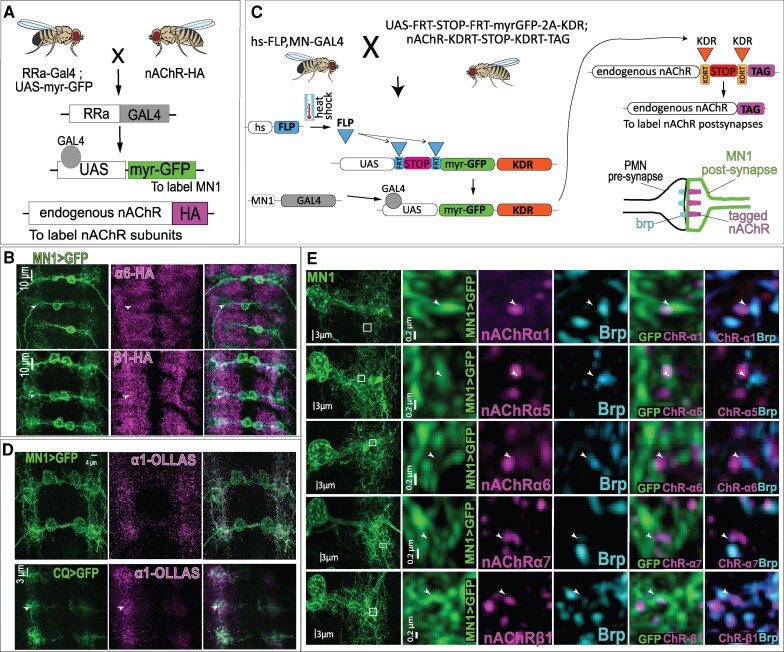
Endogenous and conditional tagging reveal synaptic localization of nAChR subunits in identified MNs. A) Strategy for labeling nAChR subunits and MN1. Endogenously HA-tagged nAChR subunits were crossed to RRa-Gal4>UAS-myr-GFP to visualize MN1 and nAChR protein. B**)** Representative confocal images showing HA-tagged α6 and β1 subunits within the dendritic arbor of MN1 labeled by myr-GFP, as shown in (A). C) Schematic of the conditional tagging strategy. Heat-shock–induced FLP recombinase excises a STOP cassette flanked by KDRT sites, allowing epitope tagging of endogenous nAChR subunits specifically in GAL4-expressing neurons. Tagged receptors are transported to postsynaptic sites in MNs. D) Representative images of conditional OLLAS-tagged α1 in MN1 (top) and in CQ-Gal4–positive MNs (bottom), with MNs labeled by myr-GFP and tagged nAChR subunits in magenta. CQ-Gal4–positive MNs (MN2, MN3, MN4, MN9, and MN10) form one-to-one NMJs with their correspondingly numbered target muscles (Fig. 1F). E) High-resolution views of MN1 dendrites showing conditional tagging of α1, α5, α6, α7, and β1 subunits (magenta) relative to the presynaptic active zone marker Bruchpilot (Brp; cyan). Tagged nAChR puncta are frequently adjacent to Brp-positive sites, consistent with postsynaptic localization. Scale bars as indicated.

To corroborate these results, we examined nAChR subunit expression in MN1 using endogenously hemagglutinin (HA)-tagged lines for α1, α6, and β1 generated in previous studies (20, 23) (Figs. 2A, B and S1). These lines were crossed to RRa-Gal4 > UAS-myr-GFP to label MN1. Numerous HA-positive puncta were observed within the MN1 dendritic arbor for all three subunits, confirming their expression at the protein level (Figs. 2A, B and S1). To further refine subunit localization in a cell-type-specific manner, we employed a conditional tagging strategy developed by Sanfilippo et al. (20), in which nAChR subunits are epitope tagged only when their endogenous alleles are transcriptionally active in the cell of interest (Fig. 2C). This approach, currently available for α1, α5, α6, and β1, revealed OLLAS epitope tag- or HA-tagged puncta in MN1 dendrites for all four subunits. These puncta were frequently adjacent to presynaptic active zones labeled by Bruchpilot (Brp), consistent with synaptic localization (Fig. 2D and E). Among these conditional lines, the α1 tag produced the most MN-restricted signal, whereas conditional tagging of α5, α6, and β1 yielded additional labeling throughout the neuropil (Figs. 2D, E and S1), likely reflecting occasional transcriptional read through of the STOP cassette and resulting tag expression outside the targeted MN population.

We next applied this conditional tagging approach using CQ-Gal4, which labels a restricted subset of MNs (five type Ib MNs per hemisegment). Consistent with the broad MN expression observed using nAChR-T2A-Gal4 lines, conditional tagging of α1, α3, and α7 confirmed their expression in CQ-positive dendrites (Figs. 2D and S1). As observed for MN1-specific tagging, the α1-OLLAS conditional line produced tagged puncta largely confined to CQ-positive MN dendrites (Figs. 2D and S1), whereas tagged α3 and α7 signals were also detected more broadly in the neuropil, again likely reflecting occasional transcriptional read through of the STOP cassette in some neurons.

Together, results from T2A-Gal4 expression analysis, endogenous protein tagging, conditional tagging, and scRNA-seq indicate that multiple nAChR subunits are expressed in larval MNs and localize to dendritic regions associated with synaptic input. These complementary approaches establish subunit expression at the level of identified MNs and provide a foundation for functional analysis of nAChR contributions to motor behavior.

### MN-specific knockdown of individual nAChR subunits alters crawling parameters

Given the widespread expression of nAChR subunits in larval MNs, we tested whether reducing subunit expression in MNs affects locomotor behavior. We used the pan-MN driver OK6-Gal4 to perform UAS-RNAi–mediated knockdown of each of the ten *Drosophila* nAChR subunits and quantified forward crawling, the primary exploratory locomotor behavior in larvae.

**Figure 3:**
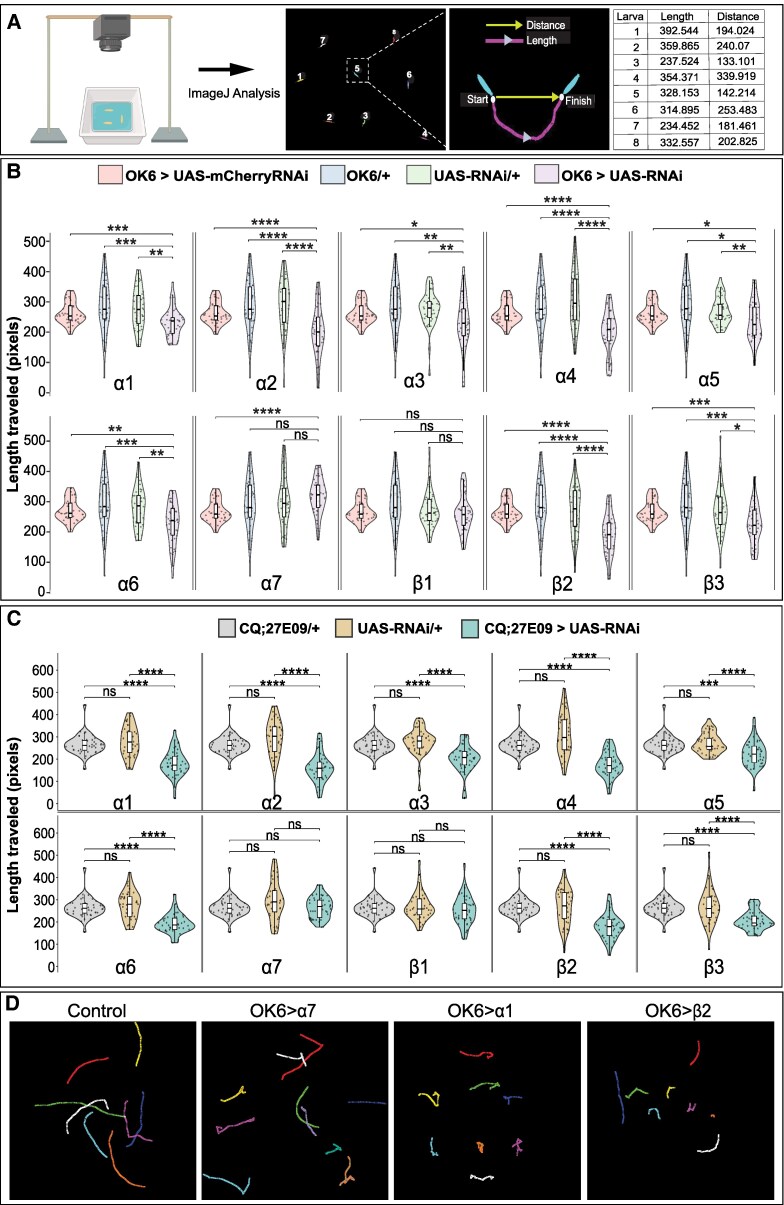
Single nAChR subunit knockdown in MNs impairs larval locomotion. A) Workflow for ImageJ and wrMTrck analysis of larval locomotion. Individual larvae were tracked to measure path length (total trajectory length). B) MN-specific knockdown of individual nAChR subunits using OK6-Gal4 (OK6-Gal4; UAS-Dicer2/UAS-RNAi) compared with three genetic controls (OK6>UAS-mCherryRNAi, OK6/+, and UAS-RNAi/+). Knockdown of α1–α6 and β2–β3 significantly reduced crawling path length. OK6>UAS-mCherryRNAi is included as a visual reference to confirm that expression of an RNAi construct alone does not affect locomotion; this control was not significantly different from the other controls. C) Knockdown of individual nAChR subunits in a subset of MNs targeted by CQ-Gal4;27E09-Gal4 produces locomotion defects largely consistent with pan-MN knockdown using OK6-Gal4 (B). The same UAS-RNAi/+ datasets were used in B and C. B, C) Pairwise statistical comparisons were performed using the Kruskal–Wallis test followed by Dunn's post hoc test with Benjamini–Hochberg correction. Brackets indicate the groups compared. Complete pairwise statistics are provided in Table S2. ns, not significant; *P < 0.05; **P < 0.01; ***P < 0.001; ****P < 0.0001. Violin plots show individual larvae (dots), with medians and interquartile ranges indicated. *n* > 39 larvae per group. Genotypes are provided in the SI Appendix. D) Representative crawling trajectories. Control larvae show normal movement; α7 knockdown larvae show no detectable impairment, α1 knockdown larvae show mild defects, and β2 knockdown larvae show severe deficits.

Forward crawling was assessed by measuring the length traveled over 60s (Fig. 3A). Knockdown of eight out of 10 subunits (α1–α6, β2, and β3) significantly reduced crawling length compared with genetic controls (Fig. 3B and Video S1). These RNAi reagents have been independently characterized in prior studies using quantitative PCR and/or immunohistochemistry, which reported reduced expression of the targeted nAChR subunits (22, 23, 38). To further address specificity, we repeated knockdown experiments for α3, α5, and α6 using independent, nonoverlapping RNAi lines, which reproduced the observed locomotor defects (Fig. S2). In contrast, knockdown of α7 and β1 did not significantly affect crawling behavior, even when tested with multiple RNAi lines (Fig. S2).

We next used a more restricted MN driver, CQ-Gal4;27E09-Gal4, which labels a defined subset of MNs, including five type Ib MNs (MN2, MN3, MN4, MN9, and MN10) and two type Is MNs per hemisegment. Knockdown of individual subunits using this driver produced patterns similar to those observed with OK6-Gal4, with significant reductions in crawling for α1–α6, β2, and β3, and little or no effect for α7 and β1 (Fig. 3C). These results indicate that altered locomotion can be observed when nAChR subunit expression is reduced in a limited subset of MNs.

**Figure 4:**
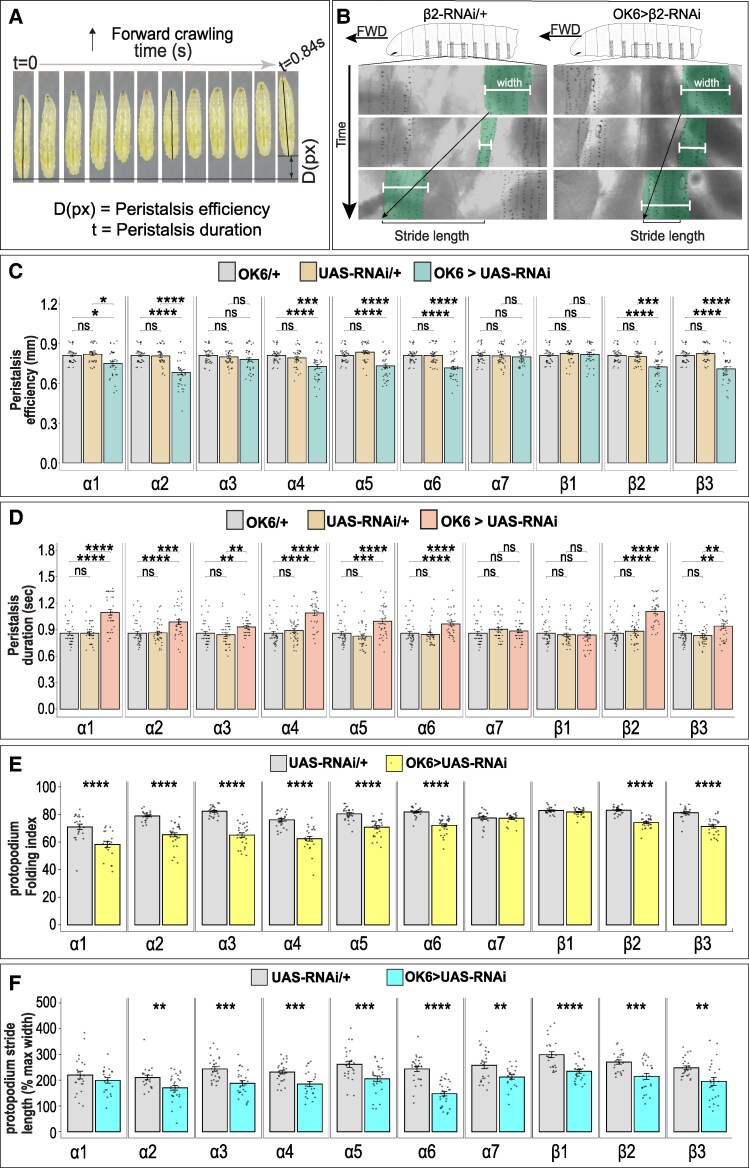
Single nAChR subunit knockdown in MNs alters crawling parameters. A) Representative time-lapse images of a single forward peristaltic wave in *Drosophila* larvae. Peristalsis efficiency is defined as the distance traveled (*D*) per peristaltic wave, and peristalsis duration as the time (*t*) required to complete one wave. B) Visualization of stride length and protopodium width in control and β2 knockdown larvae. Protopodium width (white line, labeled “width”) was measured as the anterior–posterior extent of the protopodium and is used to calculate the folding index (see E). Stride length (black brackets) indicates the displacement of the protopodium between the start and end of one peristaltic wave. β2 knockdown strongly reduces both stride length and protopodium folding. C) Knockdown of α1, α2, α4, α5, α6, β2, and β3 significantly reduces peristalsis efficiency, whereas α3, α7, and β1 knockdowns do not. D) Peristalsis duration is significantly increased following knockdown of α1, α2, α3, α4, α5, α6, β2, and β3. E) Protopodium folding index is significantly reduced following knockdown of α1, α2, α3, α4, α5, α6, β2, and β3, but is unaffected by α7 or β1 knockdowns. Protopodium width was measured using a line ROI spanning the anterior–posterior extent of a single protopodium. Folding index was calculated within each bout as: Folding index (%) = 100 × (1 − min(width)/max(width)). F) Stride length is significantly reduced following knockdown of all subunits except α1. Stride length was calculated as the displacement of the midpoint of the same width ROI between the beginning and end of each crawl bout and normalized to maximal protopodium width. In C and D, bar graphs show mean ± SEM and dots represent individual larvae. Statistical significance was assessed using the Kruskal–Wallis test followed by Dunn's multiple comparison test. In E and F, bar graphs show mean ± SEM with two to three protopodia measured per larva. Dots represent individual protopodium. Statistical significance was assessed using Student's t test. *n* > 39 larvae (C and D) and *n* > 14 larvae (E and F). ns = not significant, *P < 0.05, **P < 0.01, ***P < 0.001, ****P < 0.0001. Genotypes are provided in the SI Appendix. Complete pairwise statistics are provided in Table S2.

To further characterize these behavioral effects, we analyzed two parameters of peristaltic crawling: peristalsis efficiency (distance traveled per peristaltic wave) and peristalsis duration (time per wave). Knockdown of α1, α2, α4, α5, α6, β2, and β3 significantly reduced peristalsis efficiency, whereas α3, α7, and β1 knockdowns did not (Fig. 4C and Video S2). In addition, except for α7 and β1, peristalsis duration increased following knockdown of all other subunits (Fig. 4D and Video S2).

During crawling, larvae interact with the substrate using segmentally repeating structures termed protopodia (63, 64). Each protopodium consists of multiple rows of actin-rich denticles located in the ventroanterior region of each segment. When crawling forward, the protopodium of the contracting segment enters a swing phase, lifting from the substrate, while protopodia in other segments remain in stance. We therefore examined whether nAChR knockdown altered protopodium dynamics. The protopodium Folding index defined as 100 × (1 − folded width/resting width) was significantly reduced following knockdown of α1–α6, β2, and β3, but not α7 or β1 (Fig. 4E and Video S3). Normalized stride length (displacement per peristaltic wave) was reduced following knockdown of all subunits except α1 (Fig. 4F and Video S3).

Together, these analyses show that reducing expression of multiple nAChR subunits in MNs alters several quantitative features of larval crawling, including path length traveled, peristalsis efficiency and duration, and protopodium movement. These results indicate that several nAChR subunits contribute to optimal MN function required for normal crawling.

### Spatial organization of nAChR subunits in the neuropil and MN dendrites is associated with pair-specific functional interactions

We previously showed that individual MNs express multiple nAChR subunits and that MN-specific knockdown of single subunits is sufficient to impair locomotion. Each larval MN receives excitatory cholinergic input from multiple PMNs, giving rise to numerous postsynaptic input sites distributed along its dendritic arbor (24, 65). At each postsynaptic input site, cholinergic excitation is mediated by many pentameric nAChR channels, raising the question of whether there is spatial logic governing how different subunits are distributed across the many postsynaptic domains of MNs.

Although analysis of pairs of tagged nAChR subunits does not have the resolution to determine whether two subunits assemble into the same pentameric receptor, it can reveal whether subunits tend to co-reside within the same postsynaptic membrane domains, are enriched at distinct postsynaptic domains, or exhibit a combination of both patterns. With these considerations in mind, we asked whether particular subunit pairs preferentially co-reside within the same postsynaptic domains or are distributed across distinct postsynaptic input sites. We then examined the behavioral consequences of simultaneously knocking down subunit pairs with different degrees of spatial co-occurrence, including pairs with high, intermediate, or low overlap.

**Figure 5:**
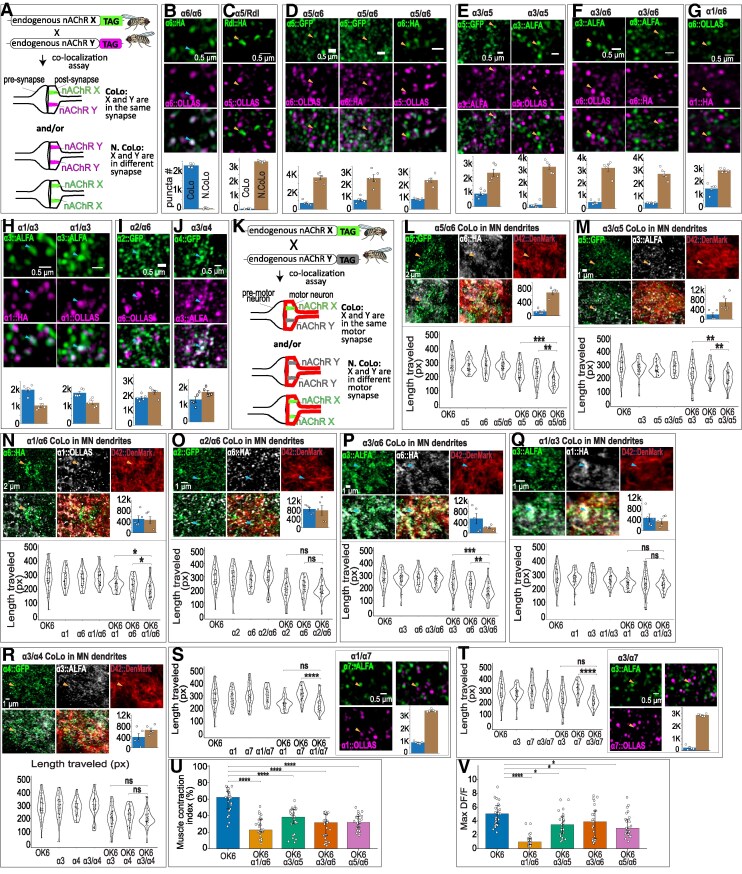
Spatial organization of nAChR subunits and pair-specific functional effects. A) Schematic of the colocalization assay used to determine whether two endogenously tagged nAChR subunits occupy the same or distinct postsynaptic membrane domains. B) Positive control for colocalization using α6::HA and α6::OLLAS, showing >98% overlap between independently tagged α6 puncta. C) Negative control for colocalization using Rdl::HA and α5::OLLAS, showing minimal overlap between GABA receptor and nAChR puncta. D–J) Pairwise colocalization of nAChR subunits in the dorsal neuropil of the VNC, including low-overlap pairs α5/α6, α3/α5, and α3/α6, high-overlap pair α1/α3, and intermediate-overlap pairs α1/α6, α2/α6, and α3/α4. Bar plots show quantification of colocalized versus noncolocalized puncta. K) Schematic of MN-restricted colocalization analysis. MN dendrites were segmented using DenMark, and only puncta overlapping MN dendritic surfaces were included in the analysis. L and M) Coocalization of α5/α6 (L) and α3/α5 (M) within MN dendrites and corresponding locomotor phenotypes. Both pairs exhibit low dendritic overlap and violin plots show enhanced defects upon MN-specific double knockdown. N) Colocalization of α1/α6 within MN dendrites and corresponding locomotor phenotype, showing intermediate overlap and a modest enhancement of defects upon double knockdown. O) Colocalization of α2/α6 within MN dendrites and corresponding locomotor phenotype, showing intermediate overlap but no enhancement of defects upon double knockdown. P) Colocalization of α3/α6 within MN dendrites and corresponding locomotor phenotype. Despite high dendritic overlap, double knockdown produces stronger defects than either single knockdown. Q) Colocalization of α1/α3 within MN dendrites and corresponding locomotor phenotype, showing high overlap and no enhancement upon double knockdown. R) Colocalization of α3/α4 within MN dendrites and corresponding locomotor phenotype, showing moderate overlap and no enhancement upon double knockdown. S and T) Colocalization of α1/α7 (S) and α3/α7 (T) in the dorsal neuropil and corresponding locomotor phenotypes. Both pairs show low overlap and no enhancement of defects upon double knockdown. For locomotor assays in L–T, each panel includes four control genotypes, two single-knockdown genotypes, and one double-knockdown genotype (seven groups total). Statistical significance was assessed using the Kruskal–Wallis test followed by Dunn's post hoc test with Benjamini–Hochberg correction (*n* > 39 larvae per genotype). All pairwise comparisons were performed, and complete statistical results are provided in Table S2; brackets indicate comparisons between double knockdowns and their corresponding single-knockdown groups. Control genotypes were not significantly different from one another in any panel. *P < 0.05, **P < 0.01, ****P < 0.0001; ns, not significant. U) Muscle contraction index was quantified by measuring changes in muscle length during forward crawling bouts in control larvae and selected double-knockdown genotypes (α1/α6, α3/α5, α3/α6, and α5/α6). The index was calculated as: Muscle contraction index (%) = 100 × (1 − min(length)/max(length)). V) Peak muscle calcium responses (Δ*F*/*F*) for the same genotypes shown in (U). In U and V, statistical significance was assessed using one-way ANOVA followed by Dunnett's post hoc test, mean ± SEM. *n* = 4–5 muscles per group. *P < 0.05, **P < 0.01, ****P < 0.0001; ns = not significant. Genotypes are provided in the SI Appendix.

To ensure the reliability of our colocalization analysis, we established thresholds using both positive and negative controls. Colocalization of the positive control, α6::HA and α6::OLLAS, showed over 98% overlap in quantified puncta, validating the sensitivity of our parameters (Fig. 5A and B). For the negative control, we analyzed overlap between Rdl::HA and α5::OLLAS. Rdl is a gamma-aminobutyric acid (GABA)-gated chloride channel, and we therefore expected minimal overlap with the nAChR subunit α5. As anticipated, colocalization between Rdl::HA and α5::OLLAS was <2%, confirming the specificity of our criteria (Fig. 5C). These controls demonstrate the robustness of our methodology for assessing colocalization between nAChR subunits.

We first examined pairwise colocalization of nAChR subunits in the dorsal neuropil of the VNC, where PMN–MN and PMN–PMN synapses are concentrated. Subunit pairs differed markedly in their degree of spatial overlap. The α5/α6 pair exhibited very low colocalization (∼17%) across multiple tag configurations (Fig. 5D), suggesting that α5- and α6-associated receptor puncta are largely segregated across postsynaptic domains. Similarly low overlap was observed for α3/α5 (∼28%) and α3/α6 (∼14%; Fig. 5E and F). α1/α3 showed high colocalization (>65%), indicating that these subunits frequently occupy overlapping postsynaptic membrane domains, while intermediate levels of overlap were observed for α1/α6 (∼36%), α2/α6 (∼45%), and α3/α4 (∼43%; Fig. 5G–J).

We next restricted colocalization analysis to MN dendrites by expressing the dendritic marker DenMark (66) to segment MN dendritic arbors and quantifying only receptor puncta that overlapped MN dendritic surfaces (Fig. 5K). For subunit pairs that were weakly overlapping in the neuropil, low colocalization generally persisted at MN dendrites. α5/α6 exhibited ∼16% colocalization within MN dendrites, and α3/α5 showed ∼22% colocalization (Fig. 5L and M), indicating strong segregation of these subunits into distinct dendritic receptor populations. Notably, MN-specific double knockdown of these segregated pairs produced locomotor phenotypes that were significantly more severe than either single knockdown (Fig. 5L and M). This suggests that segregated subunit populations likely mediate independent cholinergic inputs, both of which contribute to normal locomotion.

For subunit pairs with intermediate levels of overlap in the neuropil, the degree of spatial colocalization within MN dendrites did not consistently predict their functional interactions. For example, α1/α6 exhibited ∼51% colocalization within MN dendrites and displayed a modest enhancement of locomotor defects upon double knockdown (Fig. 5N). In contrast, α2/α6 showed similar dendritic overlap (∼52%) but no increase in phenotypic severity upon double knockdown (Fig. 5O). This indicates that from spatial overlap alone, one cannot predict if two subunits are redundant or have independent functions in MN excitation; rather, a combined interpretation of double-knockdown phenotypes along with overlap data is more informative.

Notably, α3 and α6 exhibited a striking compartment-specific pattern. Although these subunits showed very low overlap in the dorsal neuropil (∼14%), they were highly colocalized within MN dendrites (∼73%; Fig. 5F and P). Despite this high dendritic colocalization, MN-specific double knockdown of α3 and α6 produced stronger locomotor defects than either single knockdown (Fig. 5P). This indicates that receptor populations that overlap within the same postsynaptic region can nevertheless make functionally separable (i.e. not fully redundant) contributions to MN output.

In contrast, α1/α3 showed high overlap in both the neuropil (>65%) and MN dendrites (∼58%; Fig. 5H and Q), whereas α3/α4 exhibited moderate overlap in both compartments (∼43% in the neuropil and ∼38% in MN dendrites; Fig. 5J and R). For both pairs, double knockdown did not exacerbate the locomotor phenotype relative to single knockdown (Fig. 5H, J, Q, and R). This suggests that these colocalized subunits may be functionally redundant or that losing one subunit already impairs a shared synaptic process to its maximum extent.

Subunit combinations involving α7 were assayed only in the dorsal neuropil and showed low colocalization (α1/α7, 26%; α3/α7, 7%; Fig. 5S and T). Although α7 is expressed in MNs, MN-specific knockdown of α7 alone did not impair crawling behavior (Figs. 1–4). Consistent with this lack of a single-knockdown phenotype, double knockdown of α1/α7 or α3/α7 also did not intensify locomotor defects relative to α1 or α3 single knockdowns (Fig. 5S and T), indicating that α7 does not make a major detectable contribution to the MN cholinergic drive required for crawling under our experimental conditions.

Together, these data reveal that nAChR subunits are organized into distinct spatial populations across the larval motor circuit. In many cases, spatial segregation of subunits across MN dendrites is associated with stronger defects upon combined perturbation, whereas spatial co-residence can reflect either partially redundant or functionally separable contributions depending on the subunit pair. These findings suggest that distinct combinations of nAChR subunits support cholinergic signaling at different postsynaptic inputs converging onto MNs and that multiple receptor populations jointly shape motor output.

### MN-specific loss of nAChR subunits impairs muscle activation and extent of contraction

To directly assess the impact of nAChR subunit loss on motor output, we performed high-resolution muscle calcium imaging in intact larvae during forward crawling. In control larvae, peristaltic muscle contractions propagated from posterior to anterior segments, with muscles within a single segment engaging synchronously and exhibiting robust shortening, yielding an average muscle contraction index of ∼65% (Figs. 5U and S3, Video S4).

In contrast, muscle shortening was markedly reduced in nAChR double-knockdown larvae. OK6>α1/α6 larvae showed a significant reduction in contraction index to ∼20%. Intermediate reductions were observed in OK6>α3/α5, OK6>α3/α6, and OK6>α5/α6 larvae, with contraction indices of ∼35, ∼30, and ∼30%, respectively (Fig. 5U, Video S4). These decreases indicate weakened motor output and are consistent with impaired excitatory drive onto muscles following knockdown of nAChR subunits in MNs.

In addition to reduced shortening, muscle calcium responses were significantly attenuated in double-knockdown larvae. Control larvae exhibited significantly higher peak Δ*F*/*F* values compared with OK6>α1/α6, OK6>α3/α5, OK6>α3/α6, and OK6>α5/α6 larvae, indicating diminished calcium influx and reduced muscle activation under knockdown conditions (Figs. 5V and S3). Together, these findings demonstrate that nAChR subunits are required to sustain robust muscle activation and normal extent of contraction during peristaltic locomotion.

## Discussion

Our study provides a systematic characterization of the expression, subcellular localization, and functional roles of nAChR subunits within the *Drosophila* larval motor circuit. We show that MNs utilize a diverse repertoire of at least eight nAChR subunits (α1–α3, α5–α7, β1, and β2) to mediate the excitatory cholinergic drive required for locomotion. By integrating endogenous protein tagging with MN-specific functional perturbations, we demonstrate that these subunits are organized into distinct spatial populations whose combined contributions shape motor output in a pair-specific manner.

### Functional diversity and locomotor control

The requirement for multiple nAChR subunits to maintain normal crawling indicates that MNs do not rely on a single, uniform receptor population. Instead, different subunits cooperate to shape multiple aspects of motor performance, including peristaltic efficiency, timing, and protopodial dynamics. The significant reduction in crawling distance and muscle contraction amplitude observed following subunit knockdown underscores that nAChR-mediated excitation is a limiting factor for motor output.

Interestingly, although α7 and β1 are expressed and synaptically localized in MNs, their individual knockdown did not impair crawling. While this may reflect roles in behaviors not assayed here, recent proteomic evidence suggests an additional possibility: compensatory reorganization of receptor populations. Rosenthal et al. (65) showed that loss of a single nAChR subunit can trigger compensatory shifts in the abundance of other subunits at cholinergic synapses. It is therefore possible that in our MN-specific knockdowns, reduction of one subunit is partially offset by recruitment of others, preserving sufficient excitatory drive to support baseline crawling.

Consistent with context-dependent roles of nAChR subunits, recent spatial proteomic analyses revealed a marked developmental shift in nAChR composition from larval to adult stages, including downregulation of α4 and α5 and upregulation of α7, indicative of major changes in pentameric receptor assembly (65). Functionally, α7 is required for the adult escape reflex (36) but not for larval crawling, highlighting that subunit function depends on developmental stage and circuit identity. In mammals, nAChR subtype usage likewise varies across development and brain region, with distinct receptor assemblies supporting cognition and attention (66–68). Together, these parallels suggest that spatial and temporal deployment of receptor subtypes is a conserved strategy for tuning cholinergic signaling across circuits and species.

### The spatial logic of receptor distribution

A central finding of this work is that nAChR subunits are distributed across MN dendrites according to a nonuniform spatial logic, spanning a continuum from strongly segregated pairs to highly colocalized pairs. Subunit pairs with low overlap, such as α5/α6 and α3/α5, form distinct receptor populations localized to different postsynaptic domains. The enhanced locomotor defects observed upon their dual knockdown suggest that these segregated populations mediate independent cholinergic inputs, likely originating from different PMNs. Loss of both therefore removes multiple excitatory contributions to MN activity.

Rosenthal et al. (65) identified α5 and α6 as members of a homologous molecular class, subunits that had previously been linked to specific synaptic cleft organizers, including Hikaru genki (Hig) and Hasp (31, 69). The spatial segregation we observe between these subunits in the neuropil indicates that, despite their shared molecular affiliations, they are deployed into distinct postsynaptic populations that support independent cholinergic inputs.

This principle of input-specific receptor deployment has also been observed in the visual system, where distinct nAChR subunits are differentially localized to synapses formed by different cholinergic afferents onto T5 dendrites, and individual subunit perturbations alter circuit output and behavior (22). Together, these findings suggest that spatial partitioning of cholinergic receptor subtypes is a general strategy for routing excitatory drive within dendritic arbors.

In contrast, highly colocalized pairs such as α1/α3 did not show enhancement upon double knockdown. This suggests that these co-resident subunits may be functionally redundant or that losing one subunit already impairs a shared synaptic process to its maximum extent, leaving little room for additional deterioration. Supporting this interpretation, Rosenthal et al. (65) showed that deletion of α1 leads to concomitant depletion of α2 and β2 proteins, consistent with tight interdependence within shared receptor assemblies (65).

Together, these results indicate that spatial organization of nAChR subunits across MN dendrites provides an important framework for interpreting pair-specific functional interactions, while also revealing that colocalization can reflect either independent or overlapping contributions depending on subunit identity.

### From spatial arrangement to functional outcome

Our results also demonstrate that spatial proximity alone is not a perfect predictor of functional interaction. Comparison of intermediate-overlap pairs illustrates this clearly: although α1/α6 and α2/α6 exhibit nearly identical levels of dendritic colocalization (∼51–52%), only α1/α6 shows enhanced defects upon double knockdown. This indicates that from spatial overlap alone, one cannot predict whether two subunits are redundant or have independent functions in MN excitation; rather, a combined interpretation of double-knockdown phenotypes together with overlap data is more informative.

The α3/α6 pair provides an even more striking example. These subunits are largely segregated in the general neuropil but are highly co-localized within MN dendrites. Despite this high dendritic overlap, their combined knockdown produces stronger locomotor defects than either single knockdown. This demonstrates that receptor populations occupying the same postsynaptic region can nevertheless make functionally separable contributions to MN output, potentially reflecting distinct pentameric assemblies, differential biophysical properties, or engagement of separate presynaptic inputs converging onto closely spaced sites.

Similar dissociations between spatial overlap and functional redundancy have been reported in visual motion circuits, where distinct nAChR subunits exhibit complex spatial organizations—occupying either segregated or overlapping dendritic domains—to participate in different synaptic computations (21, 22). Thus, spatial organization constrains—but does not uniquely determine—functional interaction. Meaningful interpretation instead emerges from integrating localization with genetic perturbation.

By connecting dendritic receptor organization to muscle contractile force, our data establish a mechanistic link between molecular-scale receptor diversity and whole-animal behavior. The reduced peak calcium responses and contraction indices observed in double-knockdown larvae demonstrate that structured receptor populations directly influence the strength of motor output. Thus, receptor compartmentalization is not merely anatomical but has direct functional consequences for locomotion.

### Caveats and interpretation of genetic interactions

A key limitation of our approach is that RNAi-mediated knockdown is likely to produce partial loss of function. Consequently, classical genetic interpretations of nonadditivity as evidence of shared pathways or molecular complexes cannot be directly applied. Partial knockdown could mask redundancy between colocalized subunits or exaggerate apparent additivity between spatially segregated subunits.

For this reason, we do not interpret nonadditivity as proof that two subunits act within the same pentameric receptor or signaling pathway. Instead, we use spatial localization as a map for interpreting where functional interactions occur, while allowing for both redundant and nonredundant contributions within shared domains. The internal consistency of the dataset supports this framework: segregated pairs consistently show enhanced defects, highly overlapping pairs often do not, and α3/α6 demonstrates that overlap does not imply redundancy.

Future studies using MN-specific gene knockout, electrophysiology, or optical measurement of synaptic currents will be required to directly test how distinct receptor populations contribute to postsynaptic excitation.

## Conclusions

As connectomics provides the wiring diagram of the nervous system, our work adds a complementary layer of molecular tuning. We show that larval MNs deploy spatially organized receptor diversity to shape how cholinergic inputs drive motor output. Rather than assigning discrete behavioral roles to individual receptor subtypes, our findings support a model in which multiple receptor populations jointly sculpt excitation through their spatial arrangement and functional interactions.

Recent large-scale receptor mapping studies in the visual system have proposed that spatial patterns of NRs constitute a molecular cartography specifying synaptic connectivity and functional input structure (20). Our results extend this concept to a motor circuit, demonstrating that such molecular cartography is not limited to sensory processing but also governs how premotor inputs are integrated to generate behavior.

This framework should be broadly applicable to other circuits and provides a basis for understanding how receptor diversity contributes to neural computation and vulnerability in disease.

## Methods

Expression patterns of nAChR subunits were mapped in intact larvae using T2A-Gal4 lines and confirmed via immunohistochemistry with endogenously tagged subunits. MN-specific RNAi knockdown was achieved using two different MN driver lines, OK6-Gal4 and CQ-Gal4;27E09-Gal4, and the effects on larval crawling behavior were quantified using ImageJ and the wrMTrck plugin. Colocalization patterns of nAChRs in the neuropil, where MN dendrites reside, were analyzed using confocal imaging and object-based colocalization analysis in Imaris 10.0.1. Calcium imaging was conducted in intact larvae, with changes in GCaMP6s signals in muscles quantified using MATLAB code. Statistical analyses were performed using either Student's t-test or the Kruskal–Wallis test followed by Dunn's post hoc test. Significance levels are indicated as ns (not significant), *P < 0.05, **P < 0.01, and ***P < 0.001. A detailed description of reagents and methods is provided in the SI Appendix and Table S1.

## Supplementary Material

pgag173_Supplementary_Data

## Data Availability

All codes, output files, and raw, unprocessed data used in this manuscript are available at https://doi.org/10.5061/dryad.fxpnvx17f.

## Preprints

This manuscript was posted as a preprint at https://doi.org/10.1101/2025.06.07.658461.
