# Supplementary material for: Motor neurons integrate cholinergic inputs through spatial organization of diverse nicotinic receptors
Source: PNAS Nexus. 2026 May 20;5(6):pgag173. doi: 10.1093/pnasnexus/pgag173 (PMC13224740; doi:10.1093/pnasnexus/pgag173)
Supplement: pgag173_Supplementary_Data [file pgag173_supplementary_data.zip › PNASNEXUS-PNASNEXUS-2026-00120-TR-s01.pdf]

PNAS nexus

Supplementary Information for

Ankura Sitaula[1], Komal Kaur[1,2], Arianna Mogharrabi[3], Lizzy Olsen[1,4], Aref Zarin[2,4*]

[1]Biology Graduate Program, Texas A&M University, College Station, United States

[2]Department of Biology, Texas A&M University, College Station, United States

[3]Biology Undergraduate Program, Texas A&M University, College Station, United States

[4]Texas A&M Institute for Neuroscience, Texas A&M University, College Station, United States

*For correspondence: azarin@bio.tamu.edu

This PDF file includes:

Supporting text (methods)
Figure S1 to S3
Table S1 to S2
Legends for VideoS1 to S4
SI References

Other supporting materials for this manuscript include the following:

Movies S1 to S4

Supporting Information – methods

Fly culturing

Flies were maintained and reared on standard cornmeal, molasses and yeast based medium at 25 ± 1° C, ~50% humidity with a photoperiod of 12 hours light :12 hours night. For experiments involving RNAi, larvae were raised at 29 ± 1° C to increase RNAi efficiency. A complete list of fly stocks used in this study can be found in the key resource table (Tables **S1**).

Behavioral analyses and quantification

Wandering stage L3 larvae were selected from the cultured bottle and washed twice in distilled water. 6-10 larvae were then transferred to a 17 x 17 cm wide arena filled with 2% agarose using blunt forceps. Larval movement assay was adapted from a previously published paper (1). To ensure the arena surface was dry and smooth, a gentle brush was used to remove any remaining water droplets that could potentially disrupt larval locomotion. The arena was then placed under a digital camera, and following a 10 second acclimation period, the locomotor activity of the larvae was recorded for 1 minute at 30 frames per second using an iPhone mounted 30 cm from the agarose surface. Larval locomotor activity was quantified using ImageJ with the wrMTrck plugin, using parameters optimized for *Drosophila* larvae. For each 60-s video, a region of interest (ROI) was defined to restrict analysis to the area containing the larva. Larval trajectories were then extracted using wrMTrck, which outputs spatial metrics including total distance traveled and track length, corresponding to cumulative movement over time. In wrMTrck output, *distance* refers to the straight-line separation between the starting and ending positions of a trajectory (i.e., net displacement), whereas *length* corresponds to the total path traveled along the tracked trajectory (i.e., cumulative track length). Although this nomenclature differs from conventional kinematic usage, where "distance traveled" typically denotes path length and "displacement" denotes straight-line separation, we retained the wrMTrck terminology throughout the study to ensure

consistency with the software’s output variables and to avoid ambiguity when parsing and analyzing exported data files. All analyses and figure labels explicitly follow the wrMTrck definitions unless otherwise stated.

For the peristalsis efficiency and peristalsis duration assays, a two-layer 2% agarose was prepared in a 140 x 20 mm glass Petri dish. The base layer of agarose was poured into the glass and left to solidify. A groove mold was placed on top of the solidified base layer. An additional layer of agarose was then poured into the dish, covering the groove mold and allowed to solidify. The groove mold was carefully removed, leaving empty agarose grooves in the dish. The dish was placed on a sheet of paper with grids for reference. Larvae were placed into the empty agarose grooves using blunt forceps and a paintbrush. Videos were recorded using an iPhone mounted onto a ZEISS Stemi 305 microscope with similar setup as described previously (2). To evaluate peristalsis efficiency, the number of peristaltic waves required for the larvae to travel a length of 1 cm was manually counted. The length traveled per peristalsis was then determined by dividing the total length (10 mm) by the number of peristaltic waves. Peristalsis duration was assessed by manually counting the number of frames required for a peristaltic wave to propagate from the posterior body region to the anterior mouth hooks. The duration was then calculated by dividing the number of frames by the camera's frame rate to obtain time measurements.

Protopodium swing-stance assay and quantification

For the assessment of protopodia dynamics, third instar larvae were gently immobilized ventral side up on a 2% agarose gel pad to ensure consistency of orientation. A cover slip was carefully placed over the larva to maintain positioning without exerting excessive pressure. This preparation was then positioned on the stage of a confocal microscope equipped with brightfield imaging capabilities. Multiple peristalses were recorded as time series for each larva. Quantification of protopodium folding and stride length was done with custom MATLAB scripts.

Each individual protopodium consists of multiple denticle bands. Protopodium kinematics was quantified using a line ROI placed across the protopodium spanning its anterior-to-posterior denticle bands. The position and length of the line ROI were manually adjusted on a frame-by-frame basis to account for protopodium movement and changes in width during forward crawling. For each bout, the minimum and maximum widths are extracted, and folding is computed as:

Folding index (%) = 100 × (1 − min(Lengths) / max(Lengths)).

Stride length was quantified as the displacement of the midpoint of the same width ROI between the beginning and end of each forward crawling bout (displace_real). To account for differences in protopodium size across animals, stride length was normalized to the maximum protopodium width measured during the bout (displace_rate = displace_real / max(Lengths)) and is reported as a percentage of the maximum width ("% max width").

Immunohistochemistry

The late 3rd instar wandering larval brains were dissected in HL3.1 (hemolymph-like solution), fixed in 4% PFA (paraformaldehyde in 1x PBST) in a 24-well plate for 12 minutes at room temperature, and washed three times with PBST (0.3% Triton X-100 in 1x PBS) for 15 minutes each. Samples were then blocked overnight at 2 °C in PBST supplemented with 2% BSA (Fisher, BP1600-100), 1% normal donkey serum and 1% normal goat serum (Jackson ImmunoResearch Laboratories, 017-000-121 and 005-000-121). After blocking, brains were incubated in primary antibodies overnight at 2 °C. The primary antibodies were removed and washed three times in PBST for 15 minutes each. Brains were then incubated with secondary antibodies overnight at 2 °C. The secondary antibodies were removed, and the brains were washed three times in 0.3% PBST for 15 minutes each. Once washed, brains were mounted onto microscopic slides in Fluoromount-G (SouthernBiotech #0100-01) and stored at -20 °C for imaging purposes.

Primary antibodies : Rabbit anti-V5 (1:500, Cell Signaling Technology), Mouse anti-Brp/Nc82 (1:100, Developmental Studies Hybridoma Bank), Chicken anti-GFP (1:500, Invitrogen), Mouse anti-GFP (1:100, Developmental Studies Hybridoma Bank); Rabbit anti-GFP (1:500, Thermo Fisher Scientific), Mouse anti-HA (1:1000, BioLegend), Rat anti-HA (1:200, SigmaAldrich), Rat anti-OLLAS (1:100, Novus Biologicals), Rabbit anti-Dα6 (1:1000; from Mr.HAMA (3)), Atto 488 Alpaca anti-ALFA (1:500; Nanotag), Mouse anti-mCherry (1:200, Takara Bio), Rabbit anti-mCherry (1:200, Abcam), Mouse anti-ALFA (1:100, Thermo Fisher Scientific).
All secondary antibodies were purchased from Thermo Fisher Scientific and used at a working concentration of 1:200. The following antibodies were used: Alexa Fluor 405 Goat anti-Mouse, Alexa Fluor 488 Goat anti-Mouse, Alexa Fluor 488 Goat anti-Rabbit, Alexa Fluor 488 Donkey anti-Chicken, Alexa Fluor 555 Goat anti-Mouse, Alexa Fluor 555 Goat anti-Rat, Alexa Fluor 555 Goat anti-Rabbit, Alexa Fluor 594 Goat anti-Rat, Alexa Fluor 594 Goat anti-Rabbit, Alexa Fluor 647 Goat anti-Rat, Alexa Fluor 647 Donkey anti-Mouse, Alexa Fluor 647 Donkey anti-Rabbit.

Image acquisition and quantification for co-localization
Airyscan images of dissected ventral nerve cords (VNCs) from third instar wandering larvae were acquired on a Zeiss LSM900 confocal microscope equipped with a 63× oil immersion objective (NA 1.4). Imaging was performed in super-resolution (SR) mode using Zen Blue software. Raw image stacks were reconstructed using 3D Airyscan processing in Zen Blue with automatic settings. To assess spatial co-localization between pairs of nAChR subunits, object-based co-localization analysis was performed in Imaris Bitplane 10.1. For motor neuron (MN)–specific co-localization analysis, a standardized three-dimensional region of interest (ROI; 82.5 μm × 52.5 μm × 7.2 μm) was selected for each image. Within this ROI, MN dendrites and nAChR subunit puncta were segmented as separate 3D surface objects. Co-localization analysis was restricted to the MN dendritic compartment by retaining only receptor puncta that overlapped the

segmented MN dendrite surface using the Imaris “Overlapped Volume to Surfaces” filter with a control-defined overlap threshold (≥ 0.05).

Within the dendrite-associated puncta, co-localization between pairs of nAChR subunits was quantified based on physical overlap of their respective 3D surface objects. For each subunit within a given pair, dendrite-associated puncta were classified as either co-localized (overlapping puncta of the partner subtype) or non-co-localized (no overlap with the partner subtype). The proportion of co-localized puncta for each subtype was calculated as the number of co-localized puncta divided by the total number of dendrite-associated puncta for that subtype (co-localized plus non-co-localized puncta). For each receptor pair, co-localization fractions were computed separately for both assayed subunits. Because these values were nearly identical across all pairs, reflecting the same overlapping puncta population, a single representative subunit per pair was used for graphical display and statistical analysis to avoid redundancy. Importantly, this analysis quantifies spatial overlap of receptor puncta within defined neuronal compartments and does not assume co-assembly of subunits into the same receptor complex.

For dorsal neuropil measurements, a region encompassing the dorsal VNC neuropil was selected, and puncta corresponding to each fluorescently tagged nAChR subunit were segmented as separate 3D surface objects. Spatial overlap between subunit surfaces was quantified using the Imaris “Overlapped Volume to Surfaces” filter with the same control-defined overlap threshold (≥ 0.05). Puncta were classified as co-localized or non-co-localized based on whether they met this overlap criterion, and co-localization was quantified using the same proportion-based approach described for MN dendrites.

Live imaging and quantification

For live imaging, second- and third-instar larvae were washed with distilled water and placed on a 2% agarose pad positioned on a glass slide. A 2 mm x 40 mm coverslip was used to gently press the larvae into the agarose pad to immobilize them. A z-stack of the body wall was acquired using a 10x objective. For live

calcium imaging of intact muscles in third-instar larvae, multiple peristaltic cycles were recorded as a time series using a similar setup, ensuring at least 40% of the muscle area was captured during imaging.

Muscle contraction was analyzed using custom MATLAB scripts and quantified using the same approach as protopodium folding index. A linear ROI was placed on each muscle, oriented parallel to the muscle. The position and length of the ROI were manually adjusted on a frame-by-frame basis to account for changes in muscle width during forward crawling. For each crawling bout, the maximum and minimum muscle widths were extracted, and contraction index was calculated as the proportion of width reduced during contraction, defined as:

Contraction index (%) = 100 × (1 − min(width) / max(width)).

Calcium activity of individual muscles was quantified using custom MATLAB scripts. ROIs were manually placed on each muscle and adjusted on a frame-by-frame basis to track muscle movement; overlapping regions and regions not clearly in focus were avoided. Relative fluorescence changes (ΔF/F) for each muscle were calculated as $(F - F_0)/F_0$, where F_0 was defined as the tenth percentile of fluorescence values from the first one-third of the measurement.

Single-cell RNA-seq (scRNA-seq) data analysis

Publicly available scRNA-seq data were obtained from the NCBI Gene Expression Omnibus (GEO) under accession number GSE235231 (4). The raw count matrix and accompanying metadata were processed using the Seurat package (v4.3.0) in R. Transcriptional clusters were annotated with motor nerve bundle identities based on anatomical information provided in the original publication. Clusters corresponding to the same motor bundle were grouped under a unified label for visualization. Expression of nicotinic acetylcholine receptor (nAChR) subunits was analyzed across these anatomically defined groups. A DotPlot was generated to display average log-normalized expression (dot color) and the proportion of expressing cells (dot size).

Figure preparation

Images in figures were prepared as 3D projections in FIJI (ImageJ 1.54g) and assembled using Adobe Illustrator or Adobe Photoshop. Schematics were drawn in BioRender.

Statistical analysis

Statistics were performed using a combination of Microsoft Excel, MATLAB (MathWorks), R-Studio, and Python (Jupyter software). For data involving larval movement assay, peristalsis efficiency, and peristalsis duration assay Kruskal-Wallis Test with Dunn's multiple comparison was done. For data involving protopodium swing-stance assay, and muscle contraction Student's t test was done. Levels of significance were established at *: $p<.05$, **: $p<0.01$, ***: $p<0.001$, ****$P < 0.0001$. All figures depict data in bar plots or violin plots with individual values. All other pertinent information, including sample size, statistical test used, and variance can be found in the figure legends or labelled within the figure.

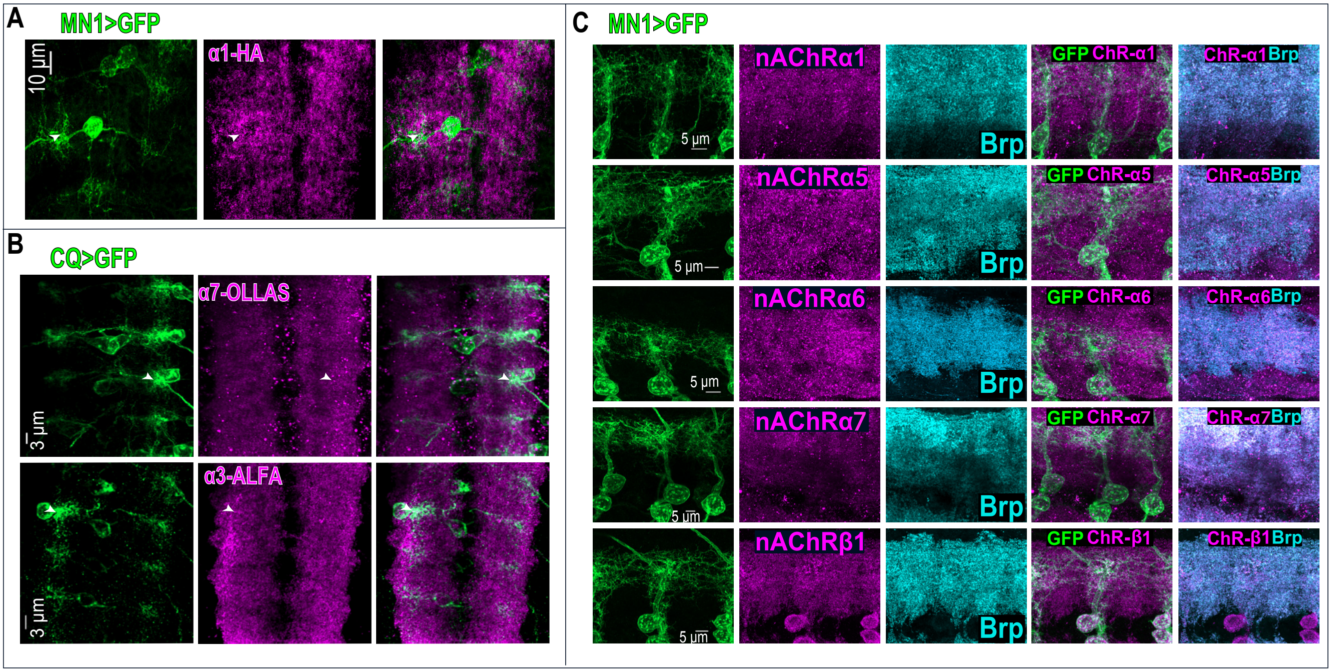

A
MN1>GFP
10 µm
α1-HA
B
CQ>GFP
α7-OLLAS
α3-ALFA
3 µm
3 µm
C
MN1>GFP
nAChRα1
Brp
GFP ChR-α1
ChR-α1 Brp
nAChRα5
GFP ChR-α5
ChR-α5 Brp
nAChRα6
GFP ChR-α6
ChR-α6 Brp
nAChRα7
GFP ChR-α7
ChR-α7 Brp
nAChRβ1
GFP ChR-β1
ChR-β1 Brp
5 µm

Supplementary Figure 1. Visualization of nAChR subunits within motor neuron dendrites.

(A) Representative confocal images showing HA-tagged α1 subunit (magenta) within the dendritic arbor of MN1 labeled by myr-GFP (green). (B) Representative images of conditional OLLAS-tagged α7 (top) and ALFA-tagged α3 (bottom) in CQ-Gal4–positive MNs, with MNs labeled by myr-GFP (green) and tagged nAChR subunits in magenta. (C) Wider field-of-view of MN1 dendrites showing conditional tagging of nAChR subunits α1, α5, α6, α7, and β1 (magenta) relative to the presynaptic active zone marker Bruchpilot (Brp; cyan). This panel corresponds to the cropped region shown in Figure 2E. Scale bars as indicated.

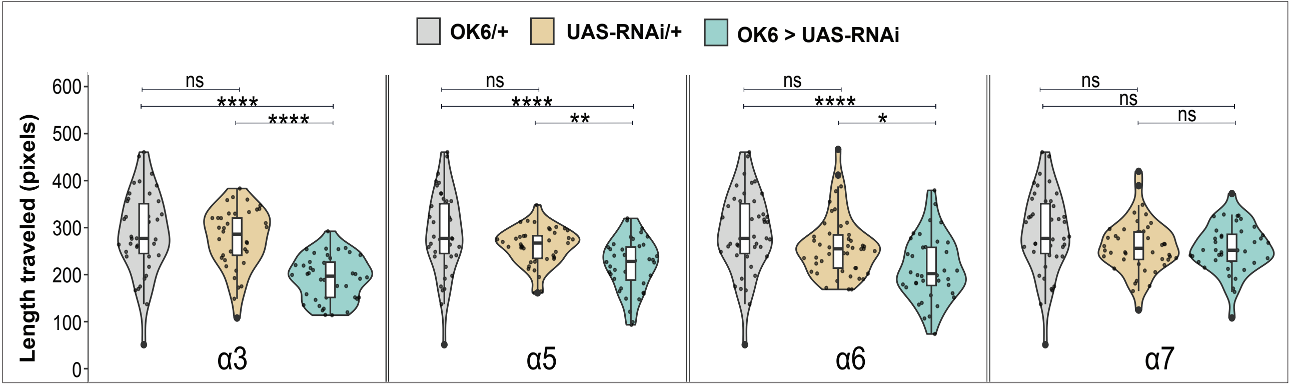

OK6/+
UAS-RNAi/+
OK6 > UAS-RNAi
Length traveled (pixels)
600
500
400
300
200
100
0
ns
****
****
ns
****
**
ns
****
*
ns
ns
ns
α3
α5
α6
α7

Supplementary Figure 2. MN-specific knockdown of the same nAChR subunits using independent RNAi constructs produces consistent behavioral outcomes, ruling out RNAi off-target effects. Quantification of total crawling path length in larvae with motor neuron (MN)-specific knockdown of nAChR subunits using OK6-Gal4;UAS-dicer/UAS-RNAi compared to OK6-Gal4;UAS-dicer/+ and UAS-RNAi/+ controls. Knockdown of α3, α5, and α6 significantly reduces locomotion, while knockdown of α7 has no observable effect on crawling behavior. These results are consistent with the findings presented in **Figure 2** of the main text, where different RNAi lines were used to selectively knock down the same subunits in MNs. Individual data points represent the crawling path length of a single larva. Data are presented as violin plots showing the distribution of path lengths, with the median and interquartile range (IQR) indicated within each plot to illustrate data variability. Statistical significance was assessed using the Kruskal-Wallis test followed by Dunn's post-hoc test (*$P < 0.05$, **$P < 0.01$, ****$P < 0.0001$, ns = non-significant). Sample sizes are $N > 39$ larvae per group.

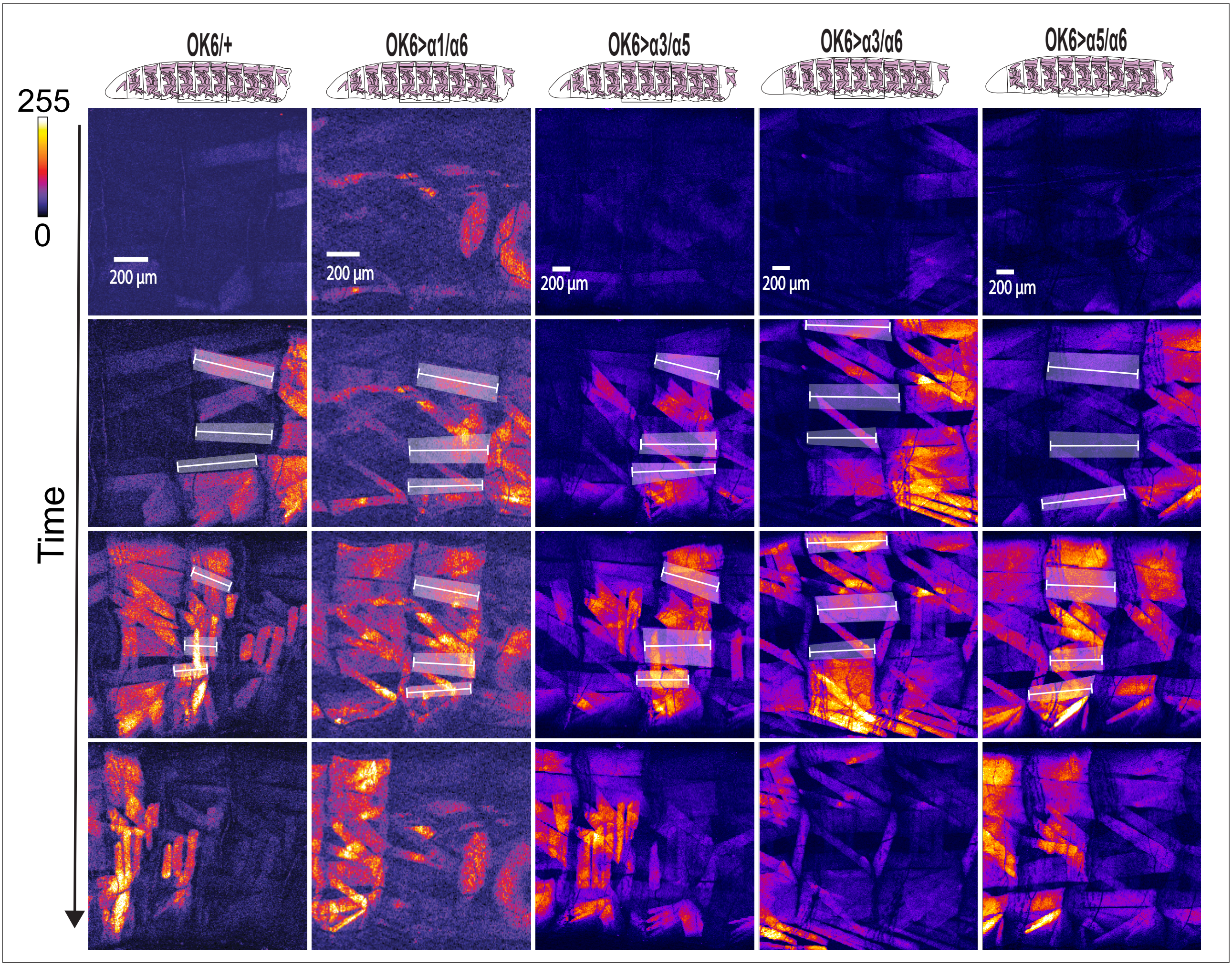
OK6/+
OK6>α1/α6
OK6>α3/α5
OK6>α3/α6
OK6>α5/α6
255
0
Time
200 µm
200 µm
200 µm
200 µm
200 µm

Supplementary Figure 3. Representative time series of muscle calcium activity (GCaMP ΔF/F) during forward crawling in control and nAChR subunit knockdown larvae. ΔF/F signals (0–255 intensity scale) are shown for OK6/+ controls and MN-specific knockdown of α1/α6, α3/α5, α3/α6, and α5/α6. Images are displayed sequentially from top to bottom, indicating progression over time. Quantification of muscle contraction index and maximum ΔF/F calcium signals for these genotypes is shown in **Fig. 5U,V** of the main text, where both measures are significantly reduced following α1/α6, α3/α5, α3/α6, or α5/α6 dual knockdown in MNs.

Tables S1: Key Resources Table

Table S2. Complete pairwise statistical comparisons for all locomotor assays. Each sheet corresponds to one figure panel. For Figures 3B, 3C, 4C, 4D, and S2, the Kruskal–Wallis test followed by Dunn's post hoc test with Benjamini–Hochberg correction was used. For Figures 4E and 4F, Student's t-test was used. For Figures 5L–T, the Kruskal–Wallis test followed by Dunn's post-hoc test with Benjamini–Hochberg correction was used. (Provided as a separate Excel file.)

Video S1. nAChR knockdown in MNs affects larval locomotion**.** Video imaging of *Dα2-RNAi* alone and *OK6-Gal4 > α2-RNAi*. Dα2 knockdown in MNs significantly impairs length traveled by larvae.

Video S2. nAChR knockdown in MNs affects peristalsis efficiency and duration. Video imaging of *Dβ2-RNAi* alone and *OK6-Gal4 > β2-RNAi*. Dβ2 knockdown in MNs affects both peristalsis efficiency and duration.

Video S3. nAChR knockdown in MNs affects stride length and protopodia folding index. Video imaging of *β2-RNAi* alone and *OK6-Gal4 > β2-RNAi*. Dβ2 knockdown in MNs affects both protopodia folding index and stride length.

Video S4. nAChR knockdown in MNs affects muscle activation and contraction. Video imaging of OK6-Gal4/+ control as compared to *OK6-Gal4 > α1/α6-RNAi, OK6-Gal4 > α3/α5-RNAi, OK6-Gal4 > α3/α6-RNAi, OK6-Gal4 > α5/α6-RNAi* knockdown impairs muscle activation and contraction.

SI References

1. D. S. Brooks, K. Vishal, J. Kawakami, S. Bouyain, E. R. Geisbrecht, Optimization of wrMTrck to monitor Drosophila larval locomotor activity. *J Insect Physiol* **93-94**, 11-17 (2016).
2. A. Sitaula, Y. Huang, A. Zarin, Application of a Dual Optogenetic Silencing-Activation Protocol to Map Motor Neurons Driving Rolling Escape Behavior in Drosophila Larvae. *Bio-protocol* **14**, e5131 (2024).
3. M. Nakayama, F. Matsushita, C. Hama, The matrix protein Hikaru genki localizes to cholinergic synaptic clefts and regulates postsynaptic organization in the Drosophila brain. *J Neurosci* **34**, 13872-13877 (2014).
4. T. H. Nguyen *et al.*, scRNA-seq data from the larval Drosophila ventral cord provides a resource for studying motor systems function and development. *Dev Cell* **59**, 1210-1230.e1219 (2024).
