# Supplementary material for: Motor neurons integrate cholinergic inputs through spatial organization of diverse nicotinic receptors
Source: PNAS Nexus. 2026 May 20;5(6):pgag173. doi: 10.1093/pnasnexus/pgag173 (PMC13224740; doi:10.1093/pnasnexus/pgag173)
Supplement: pgag173_Supplementary_Data [file pgag173_supplementary_data.zip › PNASNEXUS-PNASNEXUS-2026-00120-TR-s09.docx]

**Tables S1**: Key Resources Table

| **Reagent type (species) or resource** | **Designation** | **Source or reference** | **Additional information** |
| --- | --- | --- | --- |
| Genetic  Reagent  (*Drosophila*  *melanogaster*) | *UAS-myr-GFP; 44H10-LexA-OP-mCherry* | This study | Used in **Figure 1**. This was crossed to *T2A-Gal4* lines and MNs were imaged in intact offspring larvae. |
| Genetic reagent (*Drosophila melanogaster*) | *(Mi(1)nAChRalpha1[MI00453-TG4.0]/TM3, Sb[1] Ser[1])* | BDSC  #66780 | Used in **Figure 1**. This was crossed to *10×UAS-myr::GFP* and MNs were imaged in intact offspring larvae. |
| Genetic reagent (*Drosophila melanogaster*) | *w[*]; TI(1)CG6844[PGxRF3-1]* | (2) | Used in **Figure 1**. This was crossed to *10×UAS-myr::GFP* and MNs were imaged in intact offspring larvae. |
| Genetic reagent (*Drosophila melanogaster*) | *w[*]; TI(3)CG2302[PGxRF3-1]* | (2) | Used in **Figure 1**. This was crossed to *10×UAS-myr::GFP* and MNs were imaged in intact offspring larvae. |
| Genetic reagent (*Drosophila melanogaster*) | *y[1] w[*]; Mi(3)nAChRalpha5[MI13859-TG4.2]/SM6a* | BDSC  #76755 | Used in **Figure 1**. This was crossed to *10×UAS-myr::GFP* and MNs were imaged in intact offspring larvae. |
| Genetic reagent (*Drosophila melanogaster*) | *y[1] w[*]; Mi{Trojan-GAL4.1}(3)nAChRalpha6[MI01466-TG4.1]* | BDSC  #76137 | Used in **Figure 1**. This was crossed to *10×UAS-myr::GFP* and MNs were imaged in intact offspring larvae. |
| Genetic reagent (*Drosophila melanogaster*) | *y[1] w[*] Mi{Trojan-GAL4.1}nAChRalpha7[MI12545-TG4.1]* | BDSC  #77828 | Used in **Figure 1**. This was crossed to *10×UAS-myr::GFP* and MNs were imaged in intact offspring larvae. |
| Genetic reagent  (*Drosophila*  *Melanogaster*) | *w[*]; TI{T2A-GAL4}CG11348[RA-PGxRF3-1]* | (2) | Used in **Figure 1**. This was crossed to *10×UAS-myr::GFP* and MNs were imaged in intact offspring larvae. |
| Genetic reagent  (*Drosophila*  *Melanogaster*) | *w[*]; TI{T2A-GAL4}CG6798[RA-PGxRF3-2]/TM3* | (2) | Used in **Figure 1**. This was crossed to *10×UAS-myr::GFP* and MNs were imaged in intact offspring larvae. |
| Genetic reagent  (*Drosophila*  *Melanogaster)* | *w[*]; TI{T2A-GAL4}CG11822[PGxRF3-1]/CyO* | (2) | Used in **Figure 1**. This was crossed to *10×UAS-myr::GFP* and MNs were imaged in intact offspring larvae. |
| Genetic reagent  (*Drosophila*  *Melanogaster)* | *RRa-Gal4, UAS-myr::GFP* | This study | Used in **Figures 2, S1**. This line was crossed to endogenously tagged nAChR lines, and the VNC of offspring larvae was imaged. |
| Genetic reagent  (*Drosophila*  *Melanogaster)* | *Da1-GFP11-3xHA* | (4) | Used in **Figure S1**. This line was crossed to *RRa-Gal4, UAS-myr::GFP* and, the VNC of offspring larvae was imaged. |
| Genetic reagent  (*Drosophila*  *Melanogaster)* | *Da6-GFP11-3xHA* | (4) | Used in **Figure 2**. This line was crossed to *RRa-Gal4, UAS-myr::GFP* and, the VNC of offspring larvae was imaged. |
| Genetic reagent  (*Drosophila*  *Melanogaster)* | *w;; nAChRβ1-KDRT-smGdP-10xHA* | (3) | Used in **Figure 2**. This line was crossed to *RRa-Gal4, UAS-myr::GFP* and, the VNC of offspring larvae was imaged. |
| Genetic reagent  (*Drosophila*  *Melanogaster)* | *94G06-Gal4; UAS-FLP* | This study | Used in **Figures 2, S1**. This line was crossed to conditionally tagged nAChR lines, and the VNC of offspring larvae was imaged. |
| Genetic reagent  (*Drosophila*  *Melanogaster)* | *CQ-Gal4; UAS-FLP* | This study | Used in **Figures 2, S1**. This line was crossed to conditionally tagged nAChR lines, and the VNC of offspring larvae was imaged. |
| Genetic reagent  (*Drosophila*  *Melanogaster)* | *UAS-FRT-STOP-FRT-myrGFP-2A-KDRPEST; nAChR⍺1-KDRT-STOP-KDRT-smGdP-10xOllas* | (3) | Used in **Figures 2, S1**. This line was crossed to *94G06-Gal4; UAS-FLP or CQ-Gal4; UAS-FLP* , and the VNC of offspring larvae was imaged. |
| Genetic reagent  (*Drosophila*  *Melanogaster)* | *UAS-FRT-STOP-FRT-myrGFP-2A-KDRPEST; nAChRα3-KDRT-STOP-KDRT-1xALFA;;* | (3) | Used in **Figure S1**. This line was crossed to *CQ-Gal4; UAS-FLP* , and the VNC of offspring larvae was imaged. |
| Genetic reagent  (*Drosophila*  *Melanogaster)* | *UAS-FRT-STOP-FRT-myrGFP-2A-KDRPEST; nAChR⍺5-KDRT-STOP-KDRT-smGdP-10xOllas* | (3) | Used in **Figures 2,S1** . This line was crossed to *94G06-Gal4; UAS-FLP*, and the VNC of offspring larvae was imaged. |
| Genetic reagent  (*Drosophila*  *Melanogaster)* | *UAS-FRT-STOP-FRT-myrGFP-2A-KDRPEST; nAChR⍺6-KDRT-STOP-KDRT-smGdP-10xOllas, UAS-KDR* | (3) | Used in **Figures 2, S1**. This line was crossed to *94G06-Gal4; UAS-FLP*, and the VNC of offspring larvae was imaged. |
| Genetic reagent  (*Drosophila*  *Melanogaster)* | *UAS-FRT-STOP-FRT-myrGFP-2A-KDRPEST; nAChR⍺7-KDRT-STOP-KDRT-smGdP-10xOllas, UAS-KDR* | (3) | Used in **Figures 2,S1**. This line was crossed to *94G06-Gal4; UAS-FLP or CQ-Gal4; UAS-FLP* , and the VNC of offspring larvae was imaged. |
| Genetic reagent  (*Drosophila*  *Melanogaster)* | *UAS-FRT-STOP-FRT-myrGFP-2A-KDRPEST; nAChRβ1-KDRT-STOP-KDRT-smGdP-10xHA/TM6B* | (3) | Used in **Figures 2,S1**. This line was crossed to *94G06-Gal4; UAS-FLP*, and the VNC of offspring larvae was imaged. |
| Genetic reagent  (*Drosophila melanogaster*) | *w1118* | BDSC | Crossed as control for nAChR knockdown experiments |
| Genetic reagent (*Drosophila melanogaster*) | *OK6-Gal4* | BDSC  #64199 | MN specific nAChR knockdown. **Figure S2**. This line was crossed to *nAChR-RNAi* lines and locomotion was examined in offspring larvae. |
| Genetic reagent (*Drosophila melanogaster*) | *UAS-Dcr-2* | BDSC  #24651 | This line was crossed with *OK6-Gal4*, then with VALIUM 10 *nAChR-RNAi* lines. |
| Genetic reagent (*Drosophila melanogaster*) | *OK6-Gal4 ; UAS-Dcr-2* | This study | Used in **Figures 3-4.**This line was crossed to VALIUM 10 *nAChR-RNAi* lines and locomotion was examined in offspring larvae. |
| Genetic reagent (*Drosophila melanogaster*) | *UAS-α1-RNAi* | BDSC  #28688 | Used in **Figures 3-**4 This line was crossed to *OK6-Gal4 ; UAS-dicer* and locomotion was examined in offspring larvae. |
| Genetic reagent (*Drosophila melanogaster*) | *UAS-α2-RNAi* | BDSC  #27493 | Used in **Figures 3-4**. This line was crossed to lines *OK6-Gal4 ; UAS-dicer* and locomotion was examined in offspring larvae. |
| Genetic reagent (*Drosophila melanogaster*) | *UAS-α3-RNAi* | BDSC  #27671 | Used in **Figures 3-4**. This line was crossed to lines *OK6-Gal4 ; UAS-dicer* and locomotion was examined in offspring larvae. |
| Genetic reagent (*Drosophila melanogaster*) | *UAS-α3-RNAi* | BDSC  #61225 | Used in **Figure S2**. This line was crossed to lines *OK6-Gal4,* and locomotion was examined in offspring larvae. |
| Genetic reagent (*Drosophila melanogaster*) | *UAS-Dα4-RNAi* | BDSC  #31985 | Used in **Figures 3-4**. This line was crossed to lines *OK6-Gal4 ; UAS-dicer*, and locomotion was examined in offspring larvae. |
| Genetic reagent (*Drosophila melanogaster*) | *UAS-α5-RNAi* | BDSC  #25943 | Used in **Figures 3-4**. This line was crossed to lines *OK6-Gal4 ; UAS-dicer,* and locomotion was examined in offspring larvae. |
| Genetic reagent (*Drosophila melanogaster*) | *UAS-α5-RNAi* | BDSC  #77418 | Used in **Figure S2**. This line was crossed to lines *OK6-Gal4,* and locomotion was examined in offspring larvae. |
| Genetic reagent (*Drosophila melanogaster*) | *UAS-α6-RNAi* | BDSC  #57818 | Used in **Figure S2**. This line was crossed to lines *OK6-Gal4*, and locomotion was examined in offspring larvae. |
| Genetic reagent (*Drosophila melanogaster*) | *UAS-a6-RNAi* | BDSC  #52885 | Used in **Figures 3-4**. This line was crossed to lines *OK6-Gal4; UAS-dicer,* and locomotion was examined in offspring larvae. |
| Genetic reagent (*Drosophila melanogaster*) | *UAS-a7-RNAi* | BDSC  #27251 | Used in **Figures 3-4**. This line was crossed to lines *OK6-Gal4,* and locomotion was examined in offspring larvae. |
| Genetic reagent (*Drosophila melanogaster*) | *UAS-α7-RNAi* | BDSC  #51049 | Used in **Figure S2**. This line was crossed to lines *OK6-Gal4,* and locomotion was examined in offspring larvae. |
| Genetic reagent (*Drosophila melanogaster*) | *UAS-β1-RNAi* | BDSC  #31883 | Used in **Figures 3-4**. This line was crossed to lines *OK6-Gal4; UAS-dicer,* and locomotion was examined in offspring larvae. |
| Genetic reagent (*Drosophila melanogaster*) | *UAS-β2-RNAi* | BDSC  #28038 | Used in **Figures 3-4**. This line was crossed to lines *OK6-Gal4; UAS-dicer,* and locomotion was examined in offspring larvae. |
| Genetic reagent (*Drosophila melanogaster*) | *UAS-β3-RNAi* | BDSC  #25927 | Used in **Figures 3-4**. This line was crossed to lines *OK6-Gal4; UAS-dicer,* and locomotion was examined in offspring larvae. |
| Genetic reagent (*Drosophila melanogaster*) | *D42-Gal4 ; UAS-mCherry* | This study | Used in **Figure 5**. This line was crossed to tagged nAChR subunit pairs to perform MN specific colocalization analyses. |
| Genetic reagent (*Drosophila melanogaster*) | *α6-GFP11-3xHA*  *nAChRα6-KDRT-smGdP-10xOLLAS* | This study | Used as a control in **Figure 5** to perform co-localization analysis. |
| Genetic reagent (*Drosophila melanogaster*) | *Rdl-KDRT-smGdP-10xHA*  *nAChRα5-KDRT-smGdP-10xOLLAS* | This study | Used as a control in **Figure 5** to perform co-localization analysis. |
| Genetic reagent (*Drosophila melanogaster*) | *nAChRα5::EGFP*  *nAChRα6-KDRT-smGdP-10xOLLAS* | This study | Used in **Figure 5** to perform co-localization analysis. |
| Genetic reagent (*Drosophila melanogaster*) | *nAChRα5::EGFP*  *α6-GFP11-3xHA* | This study | Used in **Figure 5** to perform co-localization analysis. |
| Genetic reagent (*Drosophila melanogaster*) | *nAChRα5-KDRT-smGdP-10xOLLAS*  *α6-GFP11-3xHA* | This study | Used in **Figure 5** to perform co-localization analysis. |
| Genetic reagent (*Drosophila melanogaster*) | *nAChRα3-KDRT-1xALFA nAChRα5::EGFP* | This study | Used in **Figure 5** to perform co-localization analysis. |
| Genetic reagent (*Drosophila melanogaster*) | *nAChRα3-KDRT-1xALFA nAChRα5-KDRT-smGdP-10xOLLAS* | This study | Used in **Figure 5** to perform co-localization analysis. |
| Genetic reagent (*Drosophila melanogaster*) | *nAChRα3-KDRT-1xALFA*  *nAChRα6-KDRT-smGdP-10xOLLAS* | This study | Used in **Figure 5** to perform co-localization analysis. |
| Genetic reagent (*Drosophila melanogaster*) | *nAChRα3-KDRT-1xALFA*  *nAChRα6-KDRT-smGdP-10xOLLAS* | This study | Used in **Figure 5** to perform co-localization analysis. |
| Genetic reagent (*Drosophila melanogaster*) | *nAChRα6-KDRT-smGdP-10xOLLAS*  *α1-GFP11-3xHA* | This study | Used in **Figure 5** to perform co-localization analysis. |
| Genetic reagent (*Drosophila melanogaster*) | *nAChRα1-KDRT-smGdP-10xOLLAS*  *α6-GFP11-3xHA* | This study | Used in **Figure 5** to perform co-localization analysis. |
| Genetic reagent (*Drosophila melanogaster*) | *α1-GFP11-3xHA*  *nAChRα3-KDRT-1xALFA* | This study | Used in **Figure 5** to perform co-localization analysis. |
| Genetic reagent (*Drosophila melanogaster*) | *nAChRα1-KDRT-smGdP-10xOLLAS*  *nAChRα3-KDRT-1xALFA* | This study | Used in **Figure 5** to perform co-localization analysis. |
| Genetic reagent (*Drosophila melanogaster*) | *nAChRα2::EGFP*  *nAChRα6-KDRT-smGdP-10xOLLAS* | This study | Used in **Figure 5** to perform co-localization analysis. |
| Genetic reagent (*Drosophila melanogaster*) | *nAChRα2::EGFP*  *α6-GFP11-3xHA* | This study | Used in **Figure 5** to perform co-localization analysis. |
| Genetic reagent (*Drosophila melanogaster*) | *nAChRα3-KDRT-1xALFA nAChRα4::EGFP* | This study | Used in **Figure 5** to perform co-localization analysis. |
| Genetic reagent (*Drosophila melanogaster*) | *nAChRα7-KDRT-1xALFA*  *α1-GFP11-3xHA* | This study | Used in **Figure 5** to perform co-localization analysis. |
| Genetic reagent (*Drosophila melanogaster*) | *nAChRα3-KDRT-1xALFA*  *nAChRα7-KDRT-smGdP-10xOLLAS* | This study | Used in **Figure 5** to perform co-localization analysis. |
| Genetic reagent (*Drosophila melanogaster*) | *UAS-α1-RNAi ; UAS-α3-RNAi* | This study | Used in **Figure 5**. This line was crossed to lines *OK6-Gal4; UAS-dicer,* and locomotion was examined in offspring larvae. |
| Genetic reagent (*Drosophila melanogaster*) | *UAS-α1-RNAi ; UAS-α6-RNAi* | This study | Used in **Figure 5**. This line was crossed to lines *OK6-Gal4; UAS-dicer,* and locomotion was examined in offspring larvae. |
| Genetic reagent (*Drosophila melanogaster*) | *UAS-α3-RNAi ; UAS-α5-RNAi* | This study | Used in **Figure 5**. This line was crossed to lines *OK6-Gal4; UAS-dicer,* and locomotion was examined in offspring larvae. |
| Genetic reagent (*Drosophila melanogaster*) | *UAS-α3-RNAi ; UAS-α6-RNAi* | This study | Used in **Figure 5**. This line was crossed to lines *OK6-Gal4; UAS-dicer,* and locomotion was examined in offspring larvae. |
| Genetic reagent (*Drosophila melanogaster*) | *UAS-α5-RNAi ; UAS-α6-RNAi* | This study | Used in **Figure 5**. This line was crossed to lines *OK6-Gal4; UAS-dicer,* and locomotion was examined in offspring larvae. |
| Genetic reagent (*Drosophila melanogaster*) | *UAS-α1-RNAi ; UAS-α7-RNAi* | This study | Used in **Figure 5**. This line was crossed to lines *OK6-Gal4; UAS-dicer,* and locomotion was examined in offspring larvae. |
| Genetic reagent (*Drosophila melanogaster*) | *UAS-α3-RNAi ; UAS-α7-RNAi* | This study | Used in **Figure 5**. This line was crossed to lines *OK6-Gal4; UAS-dicer,* and locomotion was examined in offspring larvae. |
| Genetic reagent (*Drosophila melanogaster*) | *UAS-α3-RNAi ; UAS-α4-RNAi* | This study | Used in **Figure 5**. This line was crossed to lines *OK6-Gal4; UAS-dicer,* and locomotion was examined in offspring larvae. |
| Genetic reagent (*Drosophila melanogaster*) | *UAS-α2-RNAi ; UAS-α6-RNAi* | This study | Used in **Figure 5**. This line was crossed to lines *OK6-Gal4; UAS-dicer,* and locomotion was examined in offspring larvae. |
| Genetic reagent (*Drosophila melanogaster*) | *OK6-Gal4, UAS-Dcr-2; 44H10::GCaMP6f* | This study | Used in **Figures 5,S3**. This line was crossed with double RNAi, and the muscles of the offspring larvae were imaged. |

1. T. H. Nguyen *et al.*, scRNA-seq data from the larval Drosophila ventral cord provides a resource for studying motor systems function and development. *Dev Cell* **59**, 1210-1230.e1219 (2024).

2. S. Kondo *et al.*, Neurochemical Organization of the Drosophila Brain Visualized by Endogenously Tagged Neurotransmitter Receptors. *Cell reports* **30**, 284-297.e285 (2020).

3. P. Sanfilippo *et al.*, Mapping of multiple neurotransmitter receptor subtypes and distinct protein complexes to the connectome. *Neuron* **112**, 942-958.e913 (2024).

4. J. S. Rosenthal *et al.*, Temporal regulation of nicotinic acetylcholine receptor subunits supports central cholinergic synapse development in Drosophila. *Proc Natl Acad Sci U S A* **118** (2021).
